# Two-way Valorization of Blast Furnace Slag: Synthesis of Precipitated Calcium Carbonate and Zeolitic Heavy Metal Adsorbent

**DOI:** 10.3791/55062

**Published:** 2017-02-21

**Authors:** Evangelos Georgakopoulos, Rafael M. Santos, Yi Wai Chiang, Vasilije Manovic

**Affiliations:** ^1^Department of Offshore, Process and Energy Engineering, Cranfield University; ^2^School of Applied Chemical and Environmental Sciences, Sheridan College Institute of Technology and Advanced Learning; ^3^School of Engineering, University of Guelph; ^4^Carbon Systems Engineering, Centre for Combustion, Carbon Capture and Storage, Cranfield University

**Keywords:** Engineering, Issue 120, Blast furnace slag, waste valorization, CO_2_ utilization, mineral carbonation, hydrothermal conversion, heavy metal adsorption, precipitated calcium carbonate, zeolite

## Abstract

The aim of this work is to present a zero-waste process for storing CO_2_ in a stable and benign mineral form while producing zeolitic minerals with sufficient heavy metal adsorption capacity. To this end, blast furnace slag, a residue from iron-making, is utilized as the starting material. Calcium is selectively extracted from the slag by leaching with acetic acid (2 M CH_3_COOH) as the extraction agent. The filtered leachate is subsequently physico-chemically purified and then carbonated to form precipitated calcium carbonate (PCC) of high purity (<2 wt% non-calcium impurities, according to ICP-MS analysis). Sodium hydroxide is added to neutralize the regenerated acetate. The morphological properties of the resulting calcitic PCC are tuned for its potential application as a filler in papermaking. In parallel, the residual solids from the extraction stage are subjected to hydrothermal conversion in a caustic solution (2 M NaOH) that leads to the predominant formation of a particular zeolitic mineral phase (detected by XRD), namely analcime (NaAlSi_2_O_6_∙H_2_O). Based on its ability to adsorb Ni^2+^, as reported from batch adsorption experiments and ICP-OES analysis, this product can potentially be used in wastewater treatment or for environmental remediation applications.

**Figure:**
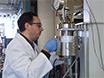


## Introduction

The indirect carbonation of industrial residues rich in alkaline metals has been widely researched as part of carbon capture and storage (CCS) technology^1^,^2^,^3^,^4^. Some amount of CO_2_ can be stored efficiently, permanently, and in a manner that is benign to the atmosphere. However, while valuable materials are formed, there is a part of the technique that remains inadequately explored. In the indirect carbonation process, calcium is selectively extracted from the material and subsequently subjected to carbonation under controlled conditions. However, the waste valorization process generates solid residues from the material; these residues are not further processed or exploited after the calcium extraction stage. Processing routes that reduce the production of such residues, or even that eliminate them, should be found. To this end, recently, there has been an effort to develop and optimize a process by which, by using blast furnace (BF) slag as the starting material, a zero-waste mineral sequestration of carbon, accompanied with the formation of useful minerals, can be achieved^5^,^6^.

Several waste materials are qualified as efficient reactants for CO_2_ mineralization. Among them, iron- and steel-making slags present considerably higher experimental CO_2_ uptakes than every other industrial waste^4^. The attractiveness of BF slag for waste valorization lies in its qualities (chemical, mineral, and morphological properties) and the potential applications of the material^5^. It is a by-product of the iron-making process, wherein impurities from iron ore are removed in a pyrometallurgical process. Based on the way it is cooled down after its separation from the molten iron, four different types of slag are generated: (i) air-cooled (*i.e.,* crystalline), (ii) granulated (*i.e.,* vitrified), (iii) expanded (*i.e.,* foamed), and (iv) pelletized.

Although the production of precipitated calcium carbonate (PCC) using the indirect carbonation of BF slag is a process that has managed to attract much attention^7^,^8^, the hydrothermal conversion of slag for the production of zeolitic minerals is a technology that has been studied and developed only during recent years^9^,^10^,^11^. However, in none of the cases has it been considered as a technique that could be used in combination with the indirect carbonation of BF slag in order to achieve the symbiotic formation of PCC and zeolites. Following the two-way valorization process herein described, these two techniques are coupled to accomplish the sufficient sequestration of CO_2_ while also obtaining zeolitic minerals and eliminating any potential solid residues. According to this procedure, CO_2_ is stored in the calcium that was extracted from slag by acid leaching via a mineral carbonation reaction^5^. To achieve the suitable PCC product properties for applications in papermaking (mineralogy, particle size distribution, and particle morphology), the leachate from the extraction stage is first physico-chemically purified^6^. In parallel, zeolitic minerals are formed in a caustic solution via the hydrothermal conversion of the solid residues resulting from the calcium extraction stage^5^.

Zeolite is an alumino-silicate mineral. It occurs naturally, but it can also be industrially produced on a large scale. Numerous unique zeolite frameworks have been identified, leading to various applications for the materials. For example, they can be used as catalysts in several industrial sectors^12^,^13^; they are found in detergents and in construction materials as additives in asphalt, concrete^14^,^15^, and Portland cement^16^,^17^; and they also have applications in the medical^18^,^19^,^20^ and agricultural^21^,^22^,^23^ domains. Furthermore, due to their large specific surface areas and their cation exchange capacities, zeolites can also be used as sorbents^24^,^25^,^26^,^27^. These particular sorbents can also be used to directly treat heavy metal-laden streams, such as wastewater or contaminated groundwater^28^,^29^,^30^,^31^. In this study, the zeolitic material produced from BF slag via the two-way valorization process is, for the first time, tested as an adsorbent for a heavy metal, namely, nickel.

For the proposed symbiotic process, an extraction agent amiable to both the PCC and zeolite formation should be used. Thus, the choice of a suitable extractant is critical. Among the several leaching agents applied in prior research on both indirect carbonation^7^,^8^ and hydrothermal conversion^10^,^11^ of BF slag, acetic acid was selected as the most promising. Hydrochloric acid^10^ exhibits detrimental effects on both the generation of PCC and on the leaching selectivity, causing significant losses in the quantities of Si and Al in the leachate solution. On the other hand, formic acid^11^ has proved to be efficient, since it manages to efficiently remove Ca and Mg from the slag while presenting remarkable leaching selectivity, leaving both the Si and the Al undisturbed. However, it presents a lower acid dissociation constant than acetic acid^33^, suggesting that the precipitation of calcium carbonate should be more readily achievable after the employment of acetate solutions as the extraction agent. It has also been shown that, in some cases, such as with the use of succinates^34^ and oxalates^35^, non-carbonate precipitates form in place of PCC. Eloneva *et al*.^36^ compared sixteen extractants for calcium removal from steelmaking slags and found acetic acid to be the most efficient (best performance between 0.5 M and 2 M extractant concentrations) and most successful (highest calcium recovery at ~100%).

The following protocol describes in detail the lab-scale experimental process that leads to the formation of high-purity PCC and a zeolitic material, with potential uses as paper fillers and heavy metal sorbents, respectively. BF slag is the starting material. The testing procedures applied for the assessment of the synthesized zeolitic material as an adequate heavy metal sorbent are also outlined.

## Protocol

### 1. Calcium Extraction from Blast Furnace Slag

NOTE: Due to the detrimental effect of acidity on leaching selectivity, the extraction of calcium takes place in two steps, using half the molarity of acetic acid (CH_3_COOH) that would be used in a single step.

1. Grind the BF slag using a mortar and pestle and sieve it to particle size below 2 mm.
2. Unseal an autoclave reactor that is equipped with a dual-impeller stirrer, a heating/cooling jacket, a pressure gauge, and a thermocouple. Make sure that the interior of the reactor vessel and the components protruding from the reactor head (stirrer shaft; impeller; and thermocouple well, which acts as a baffle) are clean and free of any impurities that may interfere with the calcium extraction process. If they are not, wash them thoroughly.
3. Weigh 100 g of the sieved BF slag (<2 mm) and place it in the vessel. Add 731 mL of CH_3_COOH (2 M) to the vessel and seal it. Make sure that the sealed reactor is properly fastened to its support.
4. Place the heating jacket in the proper position, so that it covers almost the whole vessel. Set the heating temperature to 30 °C and start mixing the slurry at 1,000 rpm. Wait until the temperature at the interior of the reactor reaches the set point (approximately 15 min), and then leave the slurry to mix at the aforementioned conditions for 60 min.
5. Once the acid extraction time has elapsed, remove the heating jacket, unseal the reactor, and pour the slurry from the reactor into a beaker. A drain valve can also be used, but the coarse solids may block the passage.
6. Vacuum-filter the slurry to separate the leachate solution from the residual solids; use filter paper with pore size of 8 µm or less. Process the solids immediately (wet cake), or leave them to dry at ambient temperature for processing at a later time. NOTE: The leachate can be stored at ambient temperature, but it should preferably be further processed (purified and carbonated) shortly afterwards to avoid the uncontrolled precipitation of dissolved compounds.
7. Wash both the reactor head and the vessel with DI water to make sure that no residue from the acid or the slag is left behind.
8. Place the dry, solid residue from the first Ca extraction step in the vessel and add 731 mL of CH_3_COOH (2 M). Repeat the same procedure (step 1.5) to mix the solids with the acid (at 30 °C and 1,000 rpm for 60 min).
9. **At the end of the second extraction step, place the post-extraction slurry into centrifuge tubes. Use large-capacity tubes (*e.g.,* 50 mL or greater) and follow standard centrifugation practices, such as ensuring equal weight in each tube.**
  1. Separate the solids from the leachate by centrifuging the slurry at 2,500 x g for a minimum of 10 min. Slowly pour the supernatant into a new bottle while keeping the solids in the tubes. NOTE: The separation of the solids (suspended silica and residual BF slag) from the calcium acetate-rich liquid phase is thus achieved.
10. Recover the solid residue resulting from the second extraction step from the tubes and re-suspend them in DI water. Perform another round of centrifugation to remove residual soluble acetates. Recover the washed solids and allow them to dry under ambient conditions.
11. Combine the solutions from both the first extraction filtration (filtrate) and the second extraction centrifugation (supernatant) to obtain the post-extraction leachate.

### 2. Physico-chemical Purification of the Post-extraction Leachate

NOTE: Despite the separation of solids from the leachate solution (step 1.9), the resulting supernatant still contains soluble or colloidal impurities. The most important of these impurities are silica, magnesium, and aluminum. According to previously published work^32^, silica solubility in pure water is proportional to the temperature of the solution (*i.e.,* by decreasing the temperature of pure water, the solubility of silica also decreases). Although the leachate solution is not pure water, it has been found that subjecting the supernatant from the solid-liquid separation of the post-extraction slurry to cooling results in further silica removal (compared to centrifugation alone)^2^. On the other hand, magnesium and aluminum impurities are present in the supernatant in the form of acetates. In order to significantly reduce their solubility, they must be transformed to insoluble metal hydroxides by pH adjustment^2^.

1. Add concentrated NaOH solution (50% w/w) to the supernatant, such that the final concentration of NaOH in the supernatant is 1.25 M; this will increase the pH to around 8.4, thus converting the magnesium and aluminum acetates to their significantly less soluble form of hydroxides. Add the caustic solution slowly while stirring and measuring the pH.
2. Place the NaOH-enriched supernatant in the refrigerator and cool it down to 1 °C to cause the extra precipitation of silica.
3. Once cooled, vacuum filter the solution using filter paper with a pore size of 0.45 µm. The micro-filtration of the solution results in the further removal of silicon and of the precipitated impurities of magnesium and aluminum.

### 3. Carbonation of the Purified Leachate

NOTE: Due to the regeneration of acetic acid upon carbonation, NaOH is used as an additive to buffer the acidity, which inhibits calcium precipitation. For the production of a purer PCC, NaOH should be used in a sub-equimolar concentration with respect to that of the CH_3_COOH used in the extraction step (2 M).

1. Pour the purified leachate into the autoclave reactor. Check both its vessel and cap components to verify that they are clean of residue from previous uses in order to avoid impurities interfering with the carbonation reactions. Add concentrated NaOH (50% w/w) to the vessel, such that the final NaOH concentration in the purified leachate solution is 1.7 M, in order to neutralize the regenerated CH_3_COOH during carbonation. Seal the reactor and carefully fasten it to its support.
2. Place the heating jacket of the reactor in the proper position. Adjust the heating temperature to 30 °C and start mixing the slurry at 1,000 rpm. Wait until the interior of the reactor reaches the desired temperature (approximately 15 min). Start the carbonation of the mixture by introducing to the reactor CO_2_ of high purity (99.5%) at 2 bar; run for 60 min.
3. At the completion of carbonation, remove the heating jacket, depressurize and unseal the reactor, and pour the carbonated slurry into a beaker. NOTE: A drain valve can also be used after depressurization, as the solids are fine.
4. Vacuum-filter the resulting slurry to separate the solid precipitates from the solution; use filter paper with a pore size of 8 µm or less. Rinse the filter cake thoroughly with DI water under vacuum to remove soluble sodium. NOTE: The marked reduction in the conductivity of the rinse filtrate can be used to confirm the rinse end-point.
5. Oven-dry the solid material at 105 °C for 24 h to retrieve the PCC.

### 4. Hydrothermal Conversion of the Extraction Solid Residues

NOTE: For hydrothermal conversion, calcium-depleted residual solids from the blast furnace slag acetic acid extraction were used. After each extraction run (including both steps), less than 50 wt% of the initial mass can be recovered (due to calcium extraction and the partial loss of colloidal silica in filtration and depending on the filter paper porosity used). Thus, multiple batches of extraction are needed to generate the mass of solids used in the hydrothermal conversion step.

1. Place 60 g of the dry, residual solids from the calcium extraction into a clean autoclave reactor. Add 300 mL of 2 M NaOH solution. Seal the reactor and fasten it to its support.
2. Place the heating jacket of the reactor in the proper position. Adjust the heating temperature to 150 °C and start mixing the slurry at 300 rpm. Wait for approximately 45-50 min, until the interior of the reactor reaches the desired temperature. Leave the slurry to mix at the aforementioned conditions for 24 h.
3. **At the completion of the hydrothermal conversion, remove the heating jacket and allow the reactor to cool down for 60 min, to approximately 35 °C. The reactor jacket's cooling circuit can also be used to speed cooling.**
  1. Unseal the reactor and pour the converted slurry into a beaker.
4. Vacuum-filter the slurry to separate the converted solids from the solution; use filter paper with a pore size of 8 µm or less. Rinse the solids thoroughly with DI water under vacuum to remove the excess caustic. NOTE: The marked reduction in conductivity of the rinse filtrate can be used to confirm the rinse end point.
5. Oven-dry the filtered material at 105 °C for 24 h to obtain the hydrothermally converted material.
6. Disaggregate the granular material using a mortar and pestle and sieve the resulting material to a particle size <0.85 mm.

### 5. Heavy Metal Adsorption Tests with the Zeolitic Product

NOTE: Ni^2+^ is selected as the heavy metal for investigation. Contaminated solutions with different initial heavy metal concentrations were synthesized. Initial heavy metal concentrations of 2-200 mg/L were chosen as appropriate for the needs of the present study.

1. To prepare the contaminated solutions for the equilibrium experiments, use a micro-pipette to add an appropriate amount of 1,000 mg/L analytical-grade standard solution of Ni^2+^ into 1 L of ultra-pure water in a volumetric flask to produce solutions of the desired Ni^2+^ concentrations (2 mg/L, 10 mg/L, 20 mg/L, 100 mg/L, and 200 mg/L).
2. In capped plastic bottles, disperse 1 g of the hydrothermally converted material resulting from step 4.6 in 100 mL of each synthetically prepared contaminated solution.
3. Add concentrated NaOH (2 M at first and 0.5 M closer to the end point) dropwise to adjust the pH of the solutions to 4-5. Continuously stir the solution at a low speed using a magnetic stirring bar on a stirring plate. Monitor the pH while adding the NaOH by using a pH electrode soaked in the solution.
4. Place the bottles in a shaker incubator and agitate them at 160 rpm and 20 °C for 24 h.
5. After mixing, add concentrated HCl (2 M at first and 0.2 M closer to the end point) dropwise to the solution to readjust the pH to 4-5. During the adjustment, continuously stir the solution at a low speed using a magnetic stirring bar on a stirring plate. Monitor the pH continuously while adding the HCl by using a pH electrode soaked in the solution.
6. Place the slurry in centrifuge tubes. Separate the solids from the solution by using a laboratory centrifuge at 2,500 x g for 5 min. Carefully pour the supernatant solution to a new bottle while keeping the solids in the centrifuge tube.
7. Acidify the solution with HNO_3_ (2 wt% nitric acid concentration) to reduce the pH to <2. NOTE: This step is performed to ensure that the ions remain in solution during storage (at ambient temperature) prior to further manipulation and analysis.
8. Determine the equilibrium concentration of the investigated heavy metal in the supernatant by using ICP-OES. NOTE: Solutions are diluted by a factor of 10-100x using 2 wt% HNO_3_ diluent, such that the expected concentration falls in the linear range of the instrument calibration (0-2 mg/L). Yttrium, at 2 mg/L, is added to each diluted sample as an internal standard. The ICP-OES instrument is operated based on the manufacturer's recommendations for the analysis of metals in wastewaters^37^. Alternate techniques for the determination of nickel concentration in solution, such as ICP-MS and AAS, are also suitable for this step.
9. Calculate the amount of heavy metal adsorbed per g of the adsorbent at equilibrium (*q_e_*) using the following formula: 
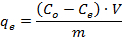
 where *C_o_* is the initial concentration (µmol/mL) of the heavy metal ions in the solution, *C_e_* is the equilibrium concentration of the heavy metal ions in the solution (µmol/mL), *V* is the volume of the contaminated solution (mL), and *m* is the mass of the dry adsorbent (g).

## Representative Results

To test the carbonate precipitates for purity and yield, several instrumental techniques can be applied. The elemental composition (including major and minor components) can be determined either by inductively coupled plasma atomic emission spectroscopy (ICP-OES), by inductively coupled plasma mass spectrometry (ICP-MS) or atomic absorption spectroscopy (AAS) following acid digestion (in HCl), or by X-ray fluorescence spectroscopy (XRF) with the sample in powder or pellet form. XRF is less sensitive for minor components (<1 wt%). More details and examples are found in De Crom *et al.*^6^ These results will demonstrate if undesired impurities are present and will help determine, by mass balance, the efficiency of converting the calcium content of the original slag into PCC. The mineral composition is best determined by X-ray powder diffraction (XRD). The resulting diffractogram provides qualitative information about the presence of crystalline mineral phases. Quantification of the relative amounts is made by Rietveld refinement technique (with an accuracy of around ± 2-3 wt%). More details and examples can be found in Santos* et al.*^38^ These results will verify if the process conditions or impurities affect the crystallization process, generating additional undesired phases besides calcite (CaCO_3_). Particle size distribution (PSD) and mean particle diameter are best determined by wet (DI water) laser diffraction. More details and examples can be found in De Crom *et al.*^6^ These results are used to assess if the PCC meets the requirements of its intended application (*i.e.,* papermaking), which usually specify an upper cut-off size and a span of distribution.

The elemental composition of the post-extraction leachate and post-carbonation products, as well as the XRD pattern and volume-based PSD of the post-carbonation precipitates, are presented in **Figures 1 **and** 2**. ICP-MS technique was used to measure the content (wt%) of certain metals (Ca, Mg, Al, and Si) in the composition of the leachate after the Ca extraction stage and before its carbonation. The use of analytical-grade acetic acid (2 M) as the leaching agent resulted in a Ca extraction of approximately 90% (**Figure 1a**). According to the results, an even higher extraction efficiency was detected for magnesium (almost 100%), another metal that can be efficiently carbonated but under more intensive conditions.

The behavior of silica and aluminum during the extraction stage was also investigated. To successfully produce alumino silicate-based zeolitic minerals through hydrothermal conversion, but also to avoid contamination of the synthesized PCC with undesired elements, both silica and aluminum should remain in the solid phase during the extraction process. According to the results, acetic acid exhibited a satisfactorily limited leaching of silica and aluminum, with almost 92% of silica and 62% of aluminum remaining unaffected during the leaching process (**Figure 1b**).

The carbonation of the purified leachate solution resulted in the production of PCC with desirable characteristics, as depicted in **Figure 2**. Based on the XRD diagram (**Figure 2b**), the mineral phase that was mainly synthesized was that of calcite (88.2 wt%), whereas small quantities of nesquehonite (Mg(HCO_3_)(OH)·2H_2_O; 3.2 wt%) and magnesian calcite (Ca_1-0.85_Mg_0-0.15_CO_3_; 2.8 wt%) were also present. From the PSD analysis of the material (**Figure 2c**), it became clear that the mean particle size was small and the particle size distribution was narrow.

In parallel with carbonation, the solid residues from the extraction stage were subjected to hydrothermal conversion. Characterization of the hydrothermally converted material, to verify the production of the zeolitic minerals and to assess the morphology, was conducted as follows. The elemental composition is most readily obtained by XRF. Trace-element determination requires acid digestion followed by ICP-OES, ICP-MS, or AAS, with the digestion carried out using sequential acid dissolution (HNO_3_-HF or HNO_3_-HClO_4_-HF) to dissolve the silica phase. While there is no specific elemental composition targeted for the converted material, this analysis helps clarify the mineral composition determined by XRD. XRD analysis, to determine mineral composition, the PSD and mean particle diameter were determined similarly to carbonate precipitates, as aforementioned. Specific surface area, pore volume, and mean pore diameter were determined by nitrogen adsorption, with the isotherms interpreted according to Brunauer-Emmett-Teller (BET) multi-point theory. Samples should be initially degassed under vacuum at 350 °C for 4 h. More details and examples can be found in Chiang *et al.*^5^

The Ca, Mg, Al, and Si content in the hydrothermally converted material, determined by using the ICP-OES technique, is shown in **Figure 3a**, whereas their mineralogical composition, determined from XRD patterns, is shown in **Figure 3b**. The mean particle size and size distribution, obtained from the PSD analysis, is shown in **Figure 3c**. The resulting material is mineralogically characterized by the presence of two main phases: analcime (NaAlSi_2_O_6_∙H_2_O) and tobermorite (Ca_5_(OH)_2_Si_6_O_16_∙4H_2_O). The existence of the latter in the converted extraction residues justifies the notable calcium content (22.5 wt%) that was detected in the chemical composition of the material, as it was analyzed using XRF. Silica (37.2 wt%) and aluminum (11.2 wt%) were the other primary elements, whereas magnesium was present in amounts of approximately 4 wt%. Based on the PSD analysis, the volume moment (De Brouckere) mean particle diameter (D[4,3]) of the converted materials was 86.6 µm, whereas the size distribution ranged from 0.594 µm to 1.11 mm. Nitrogen adsorption analysis confirmed the formation of mesoporous material (46.0 nm mean pore diameter), with the specific surface area and pore volume of the hydrothermally converted material, respectively, increasing from 4.89 m^2^/g to 95.23 m^2^/g and from 0.014 mL/g to 0.610 mL/g over the original slag.

The equilibrium adsorption isotherms of Ni^2+^ onto the hydrothermally converted material, before and after the pH adjustment of the equilibrated adsorbent-adsorbate solution, as well as the fitting of the experimental data to the linearized Langmuir, Freundlich, and Temkin adsorption models are shown in **Figure 4**.

The Langmuir model is based on some reasonable assumptions that characterize the chemisorption process. According to them, the surface of the adsorbent only offers a fixed number of adsorption sites, with identical shapes and sizes, characterized by identical adsorption capacity. The adsorbed material forms only one layer (thickness of one molecule) on the surface of the adsorbent, and the temperature is constant. Mathematically, the Langmuir model is expressed by the following equation:


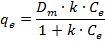


where *C_e_* is the equilibrium concentration of adsorbate in solution (µmol/100 mL), *q_e_* is the amount of metal adsorbed per g of adsorbent at equilibrium (µmol/g), *D_m_* is the theoretical maximum monolayer coverage capacity of the adsorbent (µmol/g), and *k* is the Langmuir isotherm constant (100 mL/µmol).

The Freundlich isotherm is not constrained by the assumptions required in the Langmuir model. Instead, it describes the physical adsorption process that can be applied to adsorbents with heterogeneous surfaces. The adsorption sites, distributed all over the adsorbent's surface, are characterized by different affinities for the adsorbate, whereas the adsorbed material forms more than one layer on the surface of the adsorbent. The Freundlich model is mathematically expressed as:


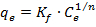


where *K_f_* and *n* are the Freundlich isotherm constants, corresponding to adsorption capacity and adsorption intensity, respectively.

Finally, the Temkin model assumes that the adsorption heat of all the molecules of the layer linearly decreases with coverage due to the adsorbent-adsorbate interactions, whereas the binding energies are uniformly distributed. The Temkin model is expressed by the following equation:


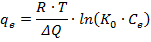


where *R* is the universal gas constant (8.314 J/mol/K), *T* is the temperature (K), *ΔQ* is the variation of adsorption energy ((J/mol)∙(g/µmol)), and *K_0_* is the Temkin isotherm equilibrium binding constant (100 mL/µmol).

The values of the coefficients for all the applied models were calculated based on the plotted adsorption isotherms (**Figure 4a**) and the linear forms of the Langmuir, Freundlich, and Temkin equations (**Figure 4b-4d**). The coefficient values, along with the linear equations, are presented in **Table 1**. Finally, comparisons between the experimental data and the theoretical adsorption isotherms of Ni^2+^ onto the activated material for the three different adsorption models are presented in **Figure 5**. Based on the contour of the graphs and the high proximity of the experimental results to the theoretical isotherm curves, it has been verified that the newly formed sorbent material can be effectively used as a Ni^2+^ adsorbent.

By comparing the fitted results presented in **Figure 5a **and** 5b**, as well as the regression coefficients (*R^2^*) for the Langmuir and Freundlich models (**Table 1**), it is clear that the Langmuir equation is the one that better describes the experimental data. This implies that the adsorption of Ni^2+^ ions on the converted material is a monolayer adsorption and that its nature is that of a chemisorption process. In order to further analyze the nature of the investigated adsorption, we also attempted to fit the Temkin model to the experimental data. From the graph shown in **Figure 5c** and its high *R^2^* (**Table 1**), it is clear that the Temkin model also fits the experimental data well. Based on the positive values of the variation of adsorption energy (*ΔQ*), it can be concluded that the adsorption is exothermic.


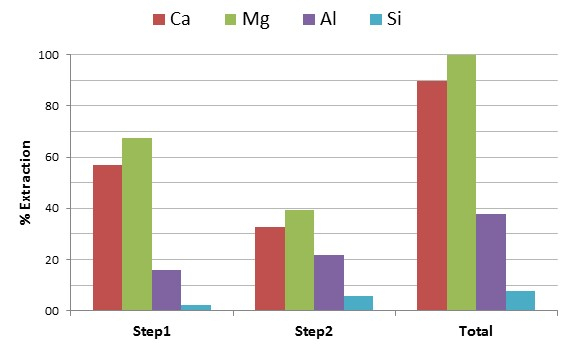
**Figure 1: Acetic acid extraction**. Concentration of Al, Ca, Mg, and Si in the leachate solutions (first step, second step, and in total) resulting from the reaction between acetic acid and ground, granulated BF slag at 30 °C, 1,000 rpm and for 60 min. Please click here to view a larger version of this figure.


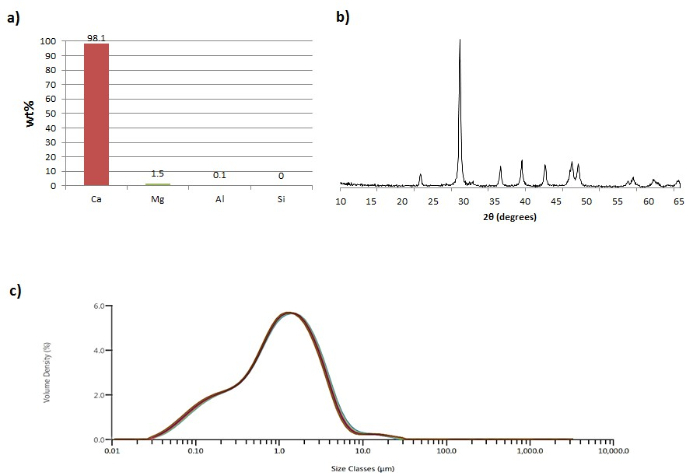
**Figure 2: Calcium carbonate precipitates. **(**a**) Composition of the carbonate precipitate, expressed in weight percentage per element, normalized to 100% total. (**b**) XRD diagram of the post-carbonation precipitate. (**c**) Particle size distribution of the post-carbonation precipitate. Reproduced from De Crom *et al.*^6^ with permission from Elsevier (3879261230348). Please click here to view a larger version of this figure.


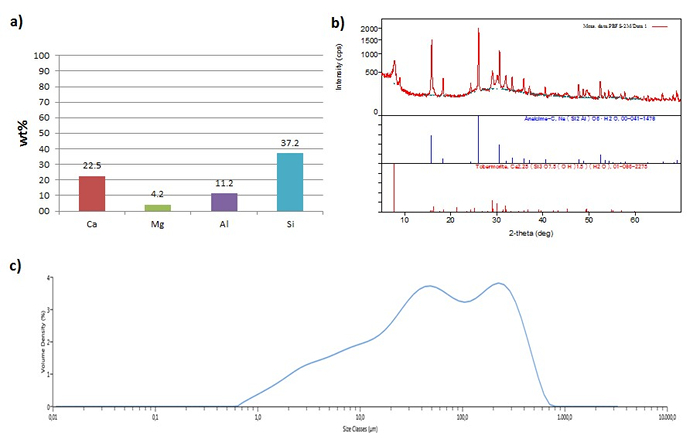
**Figure 3: Hydrothermally converted material. **(**a**) Composition of the hydrothermally converted material, expressed in weight percentage per element, normalized to 100% total. (**b**) XRD diagram of the hydrothermally converted material. (**c**) Average particle size distribution of the hydrothermally converted material. Please click here to view a larger version of this figure.


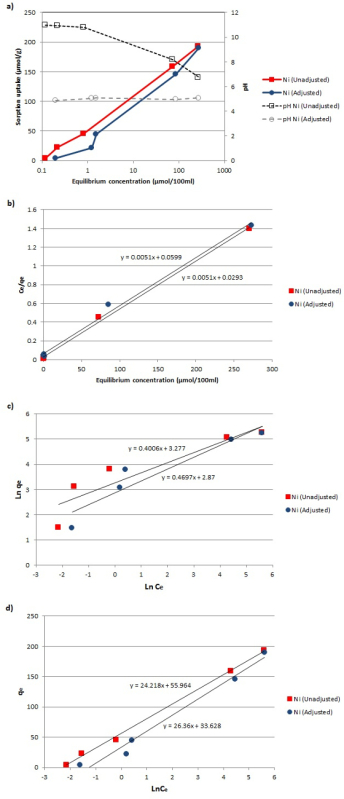
**Figure 4: Adsorption isotherms. **(**a**) Adsorption isotherm data of Ni^2+^ on the zeolitic material before and after the pH adjustment. (**b**-**d**) Fitting of the experimental data to the linearized Langmuir, Freundlich, and Temkin adsorption models. Please click here to view a larger version of this figure.


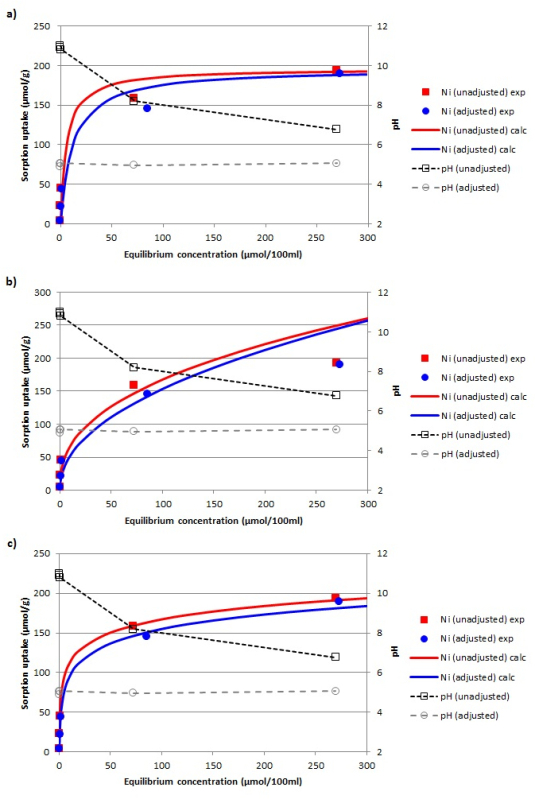
**Figure 5: Experimental and simulated data proximity. **Comparison between the experimental data (exp) and the simulated adsorption isotherms (calc) of Ni^2+^ onto the zeolitic material according to the (**a**) Langmuir, (**b**) Freundlich, and (**c**) Temkin models. Please click here to view a larger version of this figure.

**Table:** 

	**Linear Equations**	**Coefficients**	**Unadjusted**	**Adjusted**
**Langmuir Equation**	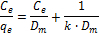	*D_m_*	196.08	196.08
*k*	0.174	0.0851
*R^2^*	0.997	0.993
**Freundlich Equation**	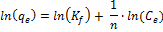	*n*	2.50	2.13
*K_f_*	26.50	17.64
*R^2^*	0.840	0.893
**Temkin Equation**	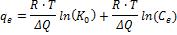	*Δ* *Q*	102.30	93.99
*K_0_*	9.97	3.58
*R^2^*	0.998	0.978

**Table 1: Adsorption isotherm parameters for the Ni^2+^ adsorption onto the zeolitic material.** Equations of, and fitted parameters from, linearized Langmuir, Freundlich, and Temkin adsorption models.

## Discussion

Although the indirect carbonation^7^,^8^ and the hydrothermal conversion^9^,^10^ of BF slags have been widely researched as separate processes, their coupling for the symbiotic synthesis of PCC and zeolitic minerals has only recently been proposed^5^, and the methodology is herein presented in detail. The most critical step of the process is the sufficient (almost total) extraction of Ca and the limited leaching of silica and aluminum from the BF slag during the extraction phase. The high amount of calcium in the leachate secures a high rate of PCC synthesis after carbonation and inhibits the generation of large amounts of undesired phases (*e.g.,* tobermorite, hydrogarnet (Ca_3_Al_2_(SiO_4_)_3-y_(OH)_4y_)) among the hydrothermally converted products^9^. On the other hand, the preservation of the greatest part of Si and Al in the post-extraction solid residues is of instrumental importance for the formation of zeolitic minerals.

To this end, among the several extractants investigated in the literature^7^,^8^,^10^,^11^,^34^,^35^,^36^, acetic acid was selected as the most suitable for the aim of this study. The particular extraction agent causes the release of high amounts of calcium from the slag to the solution, while ensuring the retention of the largest part of Si and Al in the resulting residues. This promotes the parallel formation of PCC and zeolites. The acetic acid-to-calcium molar ratio used in each extraction step was 2:1 (based on the mass of slag, the calcium content of the slag, and the volume of acetic acid solution), meaning that the total ratio over two extraction steps was 4:1. Since calcium acetate has an acetate-to-calcium ratio of 2:1, double the stoichiometric amount was used, as was found necessary by Chiang *et al.*^5^

In order to limit the presence of undesired impurities in the generated PCC, the leachate solution should be subjected to further purification before being carbonated; this is another novelty of the proposed symbiotic process. In earlier work, the PCC quality (chemical purity, mineral composition, particle size and shape) was negatively affected by impurities. For the synthesized PCC to be qualified as paper filler, certain criteria must be met. The produced PCC should be characterized by high chemical purity (min. 98 wt% Ca), homogenous mineralogical structure, small average particle size, and narrow size distribution^6^. As presented in the Representative Results section, the proposed process affords these characteristics. The precipitated carbonate is of high purity and has a calcium content of 98.1 wt% (**Figure 2a**).

The optimization of the hydrothermal conversion process resulted in the production of a material with the ability to act as a heavy metal adsorbent. The optimization was made by finding the most suitable combination of temperature, NaOH concentration, and reaction time. Tobermorite is one of the undesirable mineral phases that can form; its layered crystal structure leads to reduced specific surface area^39^, a trait important for sorbents, though it has been reported that tobermorite can act as a sorbent through an ion-exchange mechanism^40^. Nonetheless, the mineral phase that dominates the converted material in this study, under optimal conditions, is that of analcime (**Figure 3b**). It is a zeolite that has been reported to have a notable heavy metal adsorption capacity^41^,^42^ and can thus be used for the removal of toxic contaminants from wastewaters, as shown herein.

The potential use of this material as a sorbent was investigated for nickel removal from water. The pH levels of the synthetically prepared contaminated solutions of Ni^2+^ were controlled to 4-5 during the test, first, to prevent dissolution of the material in the initial acidic environment of the synthetic solution, and, second, to adjust the pH to the level typically found in heavy metal remediation conditions^43^. Three different isotherm models, namely Langmuir, Freundlich, and Temkin, were applied in order to characterize the adsorption processes (**Figures 4 **and** 5**), with the Langmuir model proving to be the most appropriate. It should be noted that the *D_m_* values attributed to the unadjusted equilibrium adsorbent-adsorbate solutions are higher than those corresponding to the equilibrium solutions after the adjustment. This is explained by the rise in pH that takes place during the adsorption reactions occurring in the solution until it reaches its equilibrium. A higher pH (>5) causes nickel to precipitate as Ni(OH)_2_, according to geochemical modeling and experimental studies by Santos *et al.*^44^, which in turn inflates the *D_m_* value. This type of heavy metal should not be accounted as the actual adsorption capacity of the tested material. In an effort to avoid such biased measurements, the pH of the equilibrated adsorbent-adsorbate solution was re-adjusted to ~5.0 by adding drops of concentrated hydrochloric acid. The lower *q_e_* values (**Figure 4a**), and consequently, the more conservative Ni adsorption estimate of the pH-adjusted solution, can thus be obtained.

The techniques described herein have the potential to be adapted to the exploitation of other materials as sources of Ca, Al, and Si for the synthesis of PCC and zeolites. Potential materials other than blast furnace slag can include steelmaking slags, incineration ashes, mining and mineral processing tailings, construction and demolition waste, natural minerals, etc. Not all of these materials contain the same proportions of Ca, Al, and Si as BF slag (which is what makes BF slag particularly attractive), but nonetheless, they can still be used to produce PCC, zeolites, or other mineral-derived products (*e.g.,* aggregates^45^ or pozzolanic materials) through similar processing techniques (some combination of extraction, precipitation and/or chemical conversion). Also, the zeolitic materials produced from BF slag or other minerals should be tested for other wastewater or remediation applications, as they likely have adsorption capacity for other heavy metals, such as Cd, Pb, and Zn^46^. Economics (the need to pay for virgin materials versus the avoidance of disposal fees for waste materials, or the financial return on utilizing the products for higher- or lower-value applications) should play a role in the identification of a suitable mineral feedstock. Substitution of other process inputs (acetic acid, sodium hydroxide, and concentrated CO_2_) by less costly or more easily recoverable alternatives should also be considered to improve processing costs.

## Disclosures

We have nothing to disclose.
